# Optogenetically engineered calcium oscillations promote autophagy-mediated cell death via AMPK activation

**DOI:** 10.1098/rsob.240001

**Published:** 2024-04-24

**Authors:** Yi-Shyun Lai, Meng-Ru Hsieh, Thi My Hang Nguyen, Ying-Chi Chen, Hsueh-Chun Wang, Wen-Tai Chiu

**Affiliations:** ^1^ Department of Biomedical Engineering, National Cheng Kung University, Tainan 701, Taiwan; ^2^ Department of Chemistry, National Cheng Kung University, Tainan 701, Taiwan; ^3^ Institute of Basic Medical Sciences, National Cheng Kung University, Tainan 701, Taiwan; ^4^ Medical Device Innovation Center, National Cheng Kung University, Tainan 701, Taiwan

**Keywords:** autophagy, calcium, optogenetics, AMPK, cell death

## Abstract

Autophagy is a double-edged sword for cells; it can lead to both cell survival and death. Calcium (Ca^2+^) signalling plays a crucial role in regulating various cellular behaviours, including cell migration, proliferation and death. In this study, we investigated the effects of modulating cytosolic Ca^2+^ levels on autophagy using chemical and optogenetic methods. Our findings revealed that ionomycin and thapsigargin induce Ca^2+^ influx to promote autophagy, whereas the Ca^2+^ chelator BAPTA-AM induces Ca^2+^ depletion and inhibits autophagy. Furthermore, the optogenetic platform allows the manipulation of illumination parameters, including density, frequency, duty cycle and duration, to create different patterns of Ca^2+^ oscillations. We used the optogenetic tool Ca^2+^-translocating channelrhodopsin, which is activated and opened by 470 nm blue light to induce Ca^2+^ influx. These results demonstrated that high-frequency Ca^2+^ oscillations induce autophagy. In addition, autophagy induction may involve Ca^2+^-activated adenosine monophosphate (AMP)-activated protein kinases. In conclusion, high-frequency optogenetic Ca^2+^ oscillations led to cell death mediated by AMP-activated protein kinase-induced autophagy.

## 1. Introduction

Autophagy (cellular self-eating) is a crucial process that occurs under both physiological and pathological conditions. Cellular autophagy is induced in pathogen infections [1,2], metabolic imbalances [3–5], intracellular damage and oxidative stress [6–8], leading to the engulfment of cytoplasmic material by a double membrane to form autophagosomes. Subsequently, autophagosomes fuse with lysosomes via the lysosomal machinery to facilitate the transport and degradation of damaged organelles, misfolded proteins and other macromolecules, followed by recycling [9–11]. Various molecular mechanisms are involved in the autophagy process, including Unc-51-like kinase 1 (ULK1) initiation complex, class III phosphatidylinositol 3-kinase complex, autophagy-related 12 conjugation system, and microtubule-associated protein 1A/1B-light chain 3 (LC3) conjugation system [12–14]. Adenosine monophosphate (AMP)-activated protein kinase (AMPK) and the mammalian target of rapamycin (mTOR) are key factors in the early stages of autophagy [15,16].

Calcium (Ca^2+^) is a versatile element that mediates a myriad of cellular physiological processes, including cell proliferation, neurotransmission, muscle contraction, cell motility, cell differentiation, apoptosis and autophagy [17–19]. At the intracellular level, a number of direct and indirect mechanisms regulate the amplitude, frequency and duty cycle of intracellular Ca^2+^ oscillations [20]. However, the extent of Ca^2+^ involvement in cellular processes may vary over time. For example, processes such as adenosine triphosphate (ATP) synthesis and muscle contraction can be induced within seconds to minutes, whereas gene regulation and cell death may require hours to days [21]. The regulation of the intracellular Ca^2+^ concentration is crucial for maintaining cellular function. Under normal conditions, the intracellular Ca^2+^ concentration (approx. 100 nM) is maintained by various regulatory mechanisms, including influx and efflux of Ca^2+^. Cells regulate the influx of external Ca^2+^ (approx. 2 mM) through Ca^2+^ channels on the cell membrane such as voltage-gated Ca^2+^ channels and the TRP superfamily. Additionally, the release of intracellular Ca^2+^ is achieved through receptors in the endoplasmic reticulum (ER), which primarily involve inositol 1,4,5-trisphosphate receptors and ryanodine receptors. These channels release Ca^2+^ from the ER lumen (approx. 100–700 μM) into the cytoplasm, thereby increasing the intracellular Ca^2+^ concentration [22,23]. When intracellular Ca^2+^ levels increase, AMPK is activated to initiate autophagy. However, the presence of a sufficient ATP/AMP ratio in mitochondria inhibits the mechanism of targeting AMPK and mTOR, consequently suppressing autophagy induction via the ULK1 axis [24–26]. Ca^2+^ can also induce death-associated protein kinase 1 (DAPK) and influence the initiation and progression of autophagy through the Beclin1-vacuolar sorting protein 34 complex [27]. Therefore, Ca^2+^ is a crucial regulatory factor that participates in multiple stages of autophagy, including the initiation, autophagosome formation and regulation of autophagic pathways. The role of Ca^2+^ in cell fate and the regulation of autophagy is pivotal [28].

Optogenetics uses light to control cellular activity. Unlike other conventional methods such as pharmacology, physics, genetics and electrophysiology, optogenetics offers higher spatial and temporal resolutions for controlling molecular activities [29]. Blue light (470 nm) is absorbed by the optogenetic molecular tool, Ca^2+^-translocating channelrhodopsin (CatCh), to control and investigate Ca^2+^ signalling in cells. Optogenetically engineered Ca^2+^ signals can be experimentally controlled to investigate the frequency, amplitude, duty cycle and duration of work. The optogenetically induced Ca^2+^ profile is an oscillating Ca^2+^ signal. Previous studies have shown that different Ca^2+^ frequencies can affect various Ca^2+^-dependent signalling pathways, including the activation of transcription factors and calpain proteases, which can lead to cell migration and death [20,30,31].

Autophagy is a double-edged sword for cell survival. Previous studies have indicated that several external stimuli can induce autophagy. However, few studies have addressed the effects of Ca^2+^ oscillations on autophagy. Therefore, optogenetics was used to investigate autophagy under different Ca^2+^ oscillation conditions. We also identified Ca^2+^ oscillations as regulators of AMPK-mediated cellular autophagy.

## 2. Material and methods

### 2.1. Cell lines

Mouse embryonic fibroblasts (MEFs), Hs 578 T cells, and CatCh-Venus-overexpressing Hs 578T (Hs 578T-CatCh-Venus) cells were maintained in Dulbecco’s modified Eagle’s medium with high-glucose (DMEM-HG; Caisson). U2OS and CatCh-Venus-overexpressing U2OS (U2OS-CatCh-Venus) cells were grown in low-glucose Dulbecco’s modified Eagle’s medium (DMEM-LG; Caisson). PANC1 and CatCh-Venus-overexpressing PANC1 (PANC1-CatCh-Venus) cells were cultured in Roswell Park Memorial Institute 1640 medium (RPMI; Caisson). All cells were supplemented with 10% fetal bovine serum (GIBCO) and 1% penicillin/streptomycin in 5% CO_2_ at 37°C.

### 2.2. Chemical reagents

Ionomycin (#I0634) and BAPTA-AM (#A1076) were purchased from Sigma–Aldrich. Thapsigargin (TG; #10522) was purchased from Cayman Chemicals. Dorsomorphin dihydrochloride (Compound C; #HY-13418), 3-methyladenine (3-MA; #HY-19312) and chloroquine (CQ; #HY-17589A) were purchased from MedChemExpress.

### 2.3. Optogenetic platform

The light illumination system (DC2100-2A; BlackRock) was controlled by a function generator to adjust the light parameters, such as power density, frequency, duty cycle and duration. The optical system included 42 high-intensity LEDs (1 W) to provide 470 nm blue light. The power density measurements were performed using a power meter (Nova II; Ophir).

### 2.4. Western blotting

Protein quantification in the collected cell lysates was performed using a DC Protein Assay Kit (#5000112; Bio-Rad) and quantified using an absorbance reader (BioTek 800 TS). The proteins were separated using 5–10% SDS-PAGE and transferred onto a nitrocellulose membrane. The membrane was blocked with 5% non-fat milk powder in Tris-buffered saline containing 0.1% Tween 20 (TBST) for 1 h or with F1 1 Min Blocking Buffer. The membrane was incubated with primary antibodies overnight at 4°C. The blots were then incubated with horseradish peroxidase-conjugated IgG secondary antibodies (#C04003 and #C04001; CROYEZ) at room temperature for 1 h. Finally, images were captured using an ECL Detection Kit (#NEL113 and #NEL112; PerkinElmer) and an Amersham Imager 600 imaging system (GE Healthcare). The primary antibodies used in Western blotting: AMPKα (#2532; Cell Signaling), phospho-AMPKα (pThr172, #2535; Cell Signaling), DAPK1 (#3008; Cell Signaling), phospho-DAP-Kinase (pSer308, #D4941; Sigma–Aldrich), mTOR (#sc-517464; Santa Cruz), phospho-mTOR (pSer2481, #sc-293089; Santa Cruz), LC3B (#TA301543; OriGene), p62 (#GTX100685; GeneTex).

### 2.5. Immunofluorescence staining

Cells were seeded in a 3 cm glass-bottom dish (#16235-1S; Alpha Plus) overnight. After light and chemical treatments, the cells were fixed with 4% paraformaldehyde for 15 min and permeabilized with 0.5% Triton X-100 for 10 min. The cells were blocked with CAS-Block^TM^ Histochemical Reagent (#00-8120; Invitrogen) for 1 h at room temperature. After CAS blocking, cells were incubated with anti-LC3 primary antibody (#PM036; MBL) overnight at 4°C. Cells were then incubated with Hoechst 33 342 (#D1306; Invitrogen) and AlexaFluor® 488- or AlexaFluor® 594-conjugated secondary antibody (#ab150077 and #ab150080; Abcam) for 1 h at room temperature. Fluorescent images were captured using an inverted fluorescence microscope (Olympus IX71). Fluorescence images were analysed using the ImageJ software (NIH).

### 2.6. Cell number

PANC1-CatCh-Venus, U2OS-CatCh-Venus and Hs 578T-CatCh-Venus cells were seeded in 3 cm dishes overnight. The cells were exposed to blue light at 470 nm (0.8 mW mm^−2^, 250 ms exposure time) for 60 min at different frequencies (0, 0.01, 0.1 or 1 Hz). After 24 and 48 h, the specimens were stained with Hoechst 33342 (#D1306; Invitrogen) for 30 min. The images were captured using an inverted wide-field fluorescence microscope (Olympus IX71). The experiments were repeated at least three times and analysed using the ImageJ software.

### 2.7. Statistical analysis

All quantitative data are presented as means ± s.e.m. ANOVA was used for the statistical analysis. SPSS Statistics 17.0 and Origin software were used to perform statistical analysis and plotting. Significance is represented as: * *p* < 0.05, ** *p* < 0.01 and *** *p* < 0.001 versus the control and ^$^ *p* < 0.05, ^$$^ *p* < 0.01 and ^$$$^ *p* < 0.001 versus the non-control groups.

## 3. Results

### 3.1. Ca^2+^-induced autophagy is AMPK-dependent

**Figure 1.**
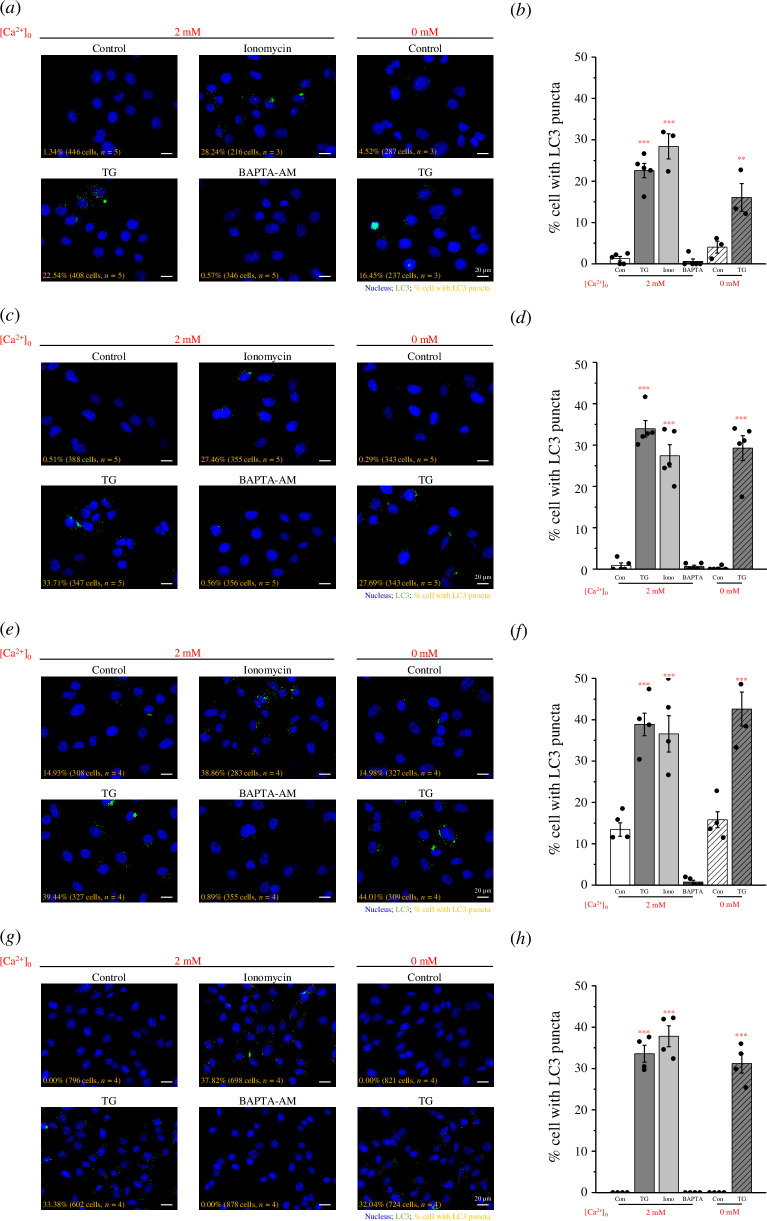
Ca^2+^-driven LC3 puncta formation. (*a*) PANC1 cells, (*c*) U2OS cells, (*e*) Hs 578 T cells and (*g*) MEFs were seeded in 3 cm glass-bottom dish overnight and treated with 2 μM ionomycin, 5 μM TG, or 20 μM BAPTA-AM in 0 and 2 mM Ca^2+^-containing media for 2 h. LC3 (green) and nuclei (blue) were observed in the fluorescence images. Scale bars: 20 μm. Quantification of the percentage of cells with LC3 puncta in (*b*) PANC1 cells, (*d*) U2OS cells, (*f)* Hs 578 T cells and (*h*) MEFs from at least three independent experiments. Data are shown as means ± s.e.m. ** *p* < 0.01, *** *p* < 0.001. MEF, mouse embryonic fibroblasts.

**Figure 2.**
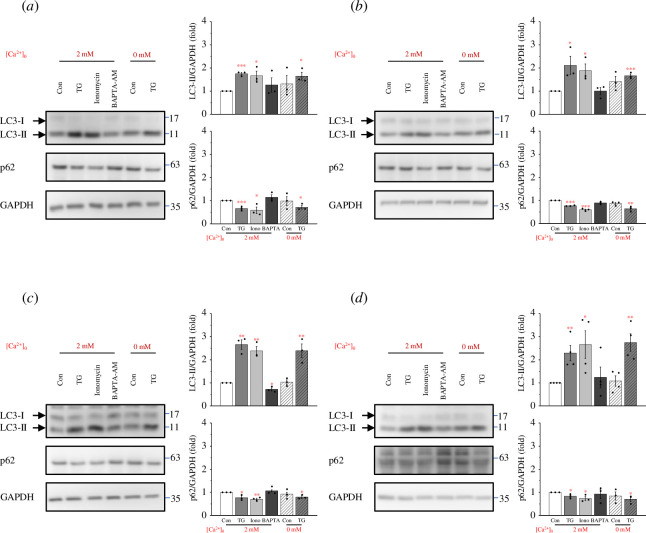
Ca^2+^ induces an increase in autophagy formation. (*a*) PANC1 cells, (*b*) U2OS cells, (*c*) Hs 578 T cells, and (*d*) MEFs were seeded in 6 cm dish overnight and treated with 2 μM ionomycin, 5 μM TG, or 20 μM BAPTA-AM in 0 or 2 mM Ca^2+^-containing media for 2 h. Cell lysates were collected, and Western blotting was performed to assess the expression levels of LC3 and p62. Glyceraldehyde 3-phosphate dehydrogenase (GAPDH) was used as an internal control. Quantification of LC3/GAPDH and p62/GAPDH ratios was conducted. Data are shown as means ± s.e.m. based on at least three independent experiments. * *p* < 0.05, ** *p* < 0.01, *** *p* < 0.001.

Autophagy is a multi-functional process that is activated under various physiological and pathological conditions. Ca^2+^ also plays a crucial role in intracellular signalling. To investigate the relationship between Ca^2+^ and autophagy, chemical stimulants were used to modulate the intracellular Ca^2+^ levels. TG is an inhibitor of sarco/endoplasmic reticulum Ca^2+^ ATPase (SERCA); it inhibits SERCA to induce Ca^2+^ release from the ER lumen, and subsequently activates store-operated Ca^2+^ entry, resulting in an increase in intracellular Ca^2+^. Ionomycin is a Ca^2+^ ionophore that forms a channel on the cell membrane, facilitating the direct transport of Ca^2+^ into intracellular regions, thereby increasing the Ca^2+^ concentration. In contrast, the Ca^2+^ chelator BAPTA-AM reduces the cytosolic Ca^2+^ concentration by chelating intracellular Ca^2+^. Four cell lines were used to verify the effect of Ca^2+^ on autophagy: pancreatic cancer PANC1 cells (figure 1*a*,*b*), osteosarcoma U2OS cells (figure 1*c*,*d*), breast cancer Hs 578 T cells (figure 1*e*,*f*) and MEFs (figure 1*g*,*h*). LC3 was used as a marker of autophagy in cells. In the resting state without any stimulation, PANC1, U2OS and MEFs did not undergo autophagy (less than 2%; figure 1*b*,*d,h*). In contrast to these three cell lines, Hs 578 T cells, had a higher proportion of cells undergoing autophagy (15%; figure 1*f*). In the presence of 2 mM Ca^2+^, both TG and ionomycin significantly induced autophagy in cells; however, there was no significant difference in the degree of autophagy induced by the two chemicals (figure 1*b*,*d*,*f,h*). In Hs 578 T cells, BAPTA-AM almost completely inhibited autophagy (less than 1%; figure 1*f*). In the absence of Ca^2+^ in the culture medium (0 mM Ca^2+^), TG also significantly induced cell autophagy, and the effect was not statistically different from that in 2 mM Ca^2+^ culture medium (figure 1*b*,*d*,*f,h*). Furthermore, we found that the expression levels of LC3-II increased while the expression levels of p62 decreased upon the addition of TG or ionomycin to increase cytosolic Ca^2+^. In contrast, the expression levels of LC3-II and p62 did not change significantly when BAPTA-AM was added to chelate cytosolic Ca^2+^. In addition, even in a Ca^2+^-free buffer, the increase in cytosolic Ca^2+^ caused by TG also caused an increase in LC3-II expression and a decrease in p62 expression (figure 2). Hence, Ca^2+^ has the ability to induce autophagy.

**Figure 3.**
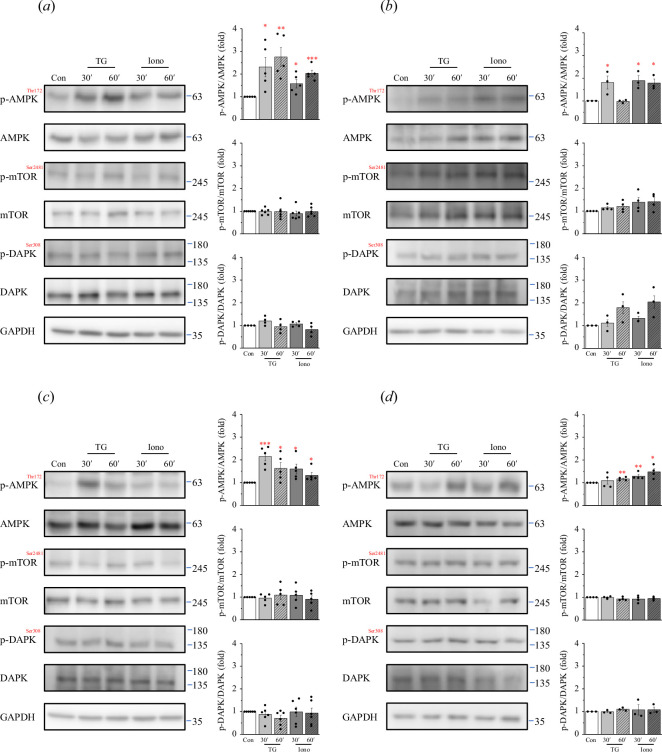
Ca^2+^ induces AMPK activation. (*a*) PANC1 cells, (*b*) U2OS cells, (*c*) Hs 578 T cells and (*d*) MEFs were treated with 2 μM ionomycin and 5 μM TG for 30 and 60 min. Cell lysates were collected, and Western blotting was performed to assess the expression levels of p-AMPK, AMPK, p-DAPK, DAPK, p-mTOR and mTOR. GAPDH was used as an internal control. Quantification of p-AMPK/AMPK, p-DAPK/DAPK, and p-mTOR/mTOR levels was conducted. Data are shown as means ± s.e.m. based on at least three independent experiments. * *p* < 0.05, ** *p* < 0.01, *** *p* < 0.001. AMPK, AMP-activated protein kinase; DAPK, death-associated protein kinase; MEF, mouse embryonic fibroblasts; mTOR, mammalian target of rapamycin.

Ca^2+^ is involved in various signalling pathways associated with autophagy, such as AMPK, mTOR and DAPK signalling [25,26]. Treatment with ionomycin and TG, which induced intracellular Ca^2+^ increase, led to the formation of LC3 puncta. Interestingly, we observed that ionomycin and TG treatment AMPK increased the phosphorylation and activation in all four cell lines. However, they did not increase the phosphorylation or activation of mTOR and DAPK in these cells (figure 3). Thus, Ca^2+^ activates AMPK, leading to autophagy.

### 3.2. Optogenetically engineered Ca^2+^ oscillations regulated autophagy

**Figure 4.**
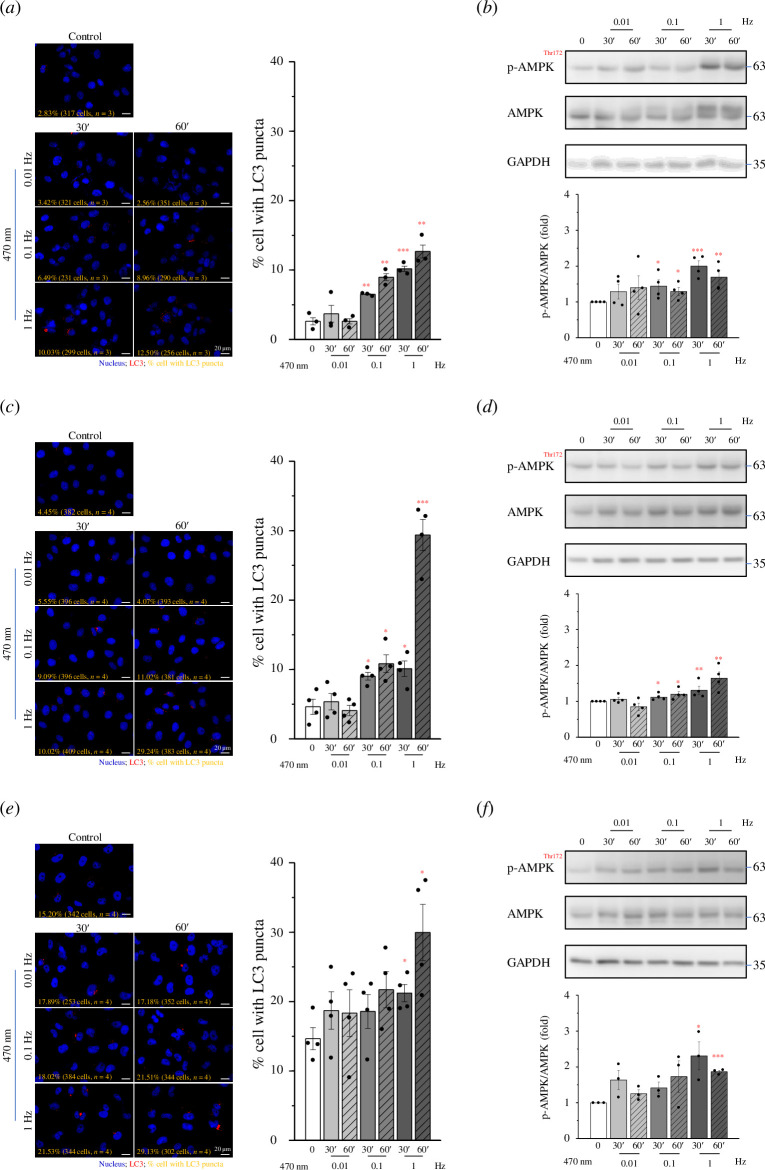
High-frequency Ca^2+^ oscillation promotes cellular autophagy. The cells were continuously exposed to blue light at 470 nm (0.8 mW mm^−2^, 250 ms) at varying frequencies (0.01, 0.1 and 1 Hz) for 30 and 60 min. Fluorescence images of (*a*) PANC1-CatCh-Venus, (*c*) U2OS-CatCh-Venus and (*e*) Hs 578T-CatCh-Venus cells showing LC3 (green) and nuclei (blue) staining along with the percentage of cells with LC3 puncta formation. Scale bars: 20 μm. Western blotting was performed to examine the phosphorylation and expression of AMPK, p-AMPK and GAPDH in (*b*) PANC1-CatCh-Venus, (*d*) U2OS-CatCh-Venus and (*f*) Hs 578T-CatCh-Venus cells. Data are shown as means ± s.e.m. based on at least three independent experiments. * *p* < 0.05, ** *p* < 0.01, *** *p* < 0.001. AMP, adenosine monophosphate; AMPK, AMP-activated protein kinase.

To evaluate the impact of Ca^2+^ oscillations on cellular autophagy, CatCh was overexpressed in cells to engage in the optogenetic manipulation of specific Ca^2+^ signals. It has been previously demonstrated that the light-activatable CatCh Ca^2+^ channel induces Ca^2+^ influx through activation using 470 nm blue light. When parental U2OS cells were exposed to 470 nm blue light, there was no increase in LC3 puncta, indicating that the absence of CatCh expression did not induce Ca^2+^ influx or subsequent autophagy (see electronic supplementary material, figure S1). Therefore, we overexpressed CatCh in the PANC1, U2OS and Hs 578 T cell lines. Optogenetic blue light stimulation (at the intensity of 0.8 mW mm^−2^ with 250 ms exposure time) of PANC1 and U2OS cell lines expressing CatCh displayed an increase in LC3 puncta at 0.1 and 1 Hz stimulation frequencies (figure 4*a,c*). However, an increase in LC3 puncta was observed only at a stimulation frequency of up to 1 Hz in Hs 578T-CatCh-Venus cells (figure 4*e*). This suggests that a substantial influx of Ca^2+^ is necessary to induce autophagy. Consequently, we examined the pathways involved in optogenetically engineered Ca^2+^-induced autophagy by Western blotting. Under optogenetic stimulation, we observed that the phosphorylation and activation of AMPK increased with high-frequency Ca^2+^ oscillations (figure 4*b*,*d,f*), whereas there were no changes in the levels of mTOR or DAPK under the same stimulation conditions (see electronic supplementary material, figure S2). In addition, we found that optogenetically induced autophagy positively correlated with AMPK phosphorylation and activation. These findings suggest that Ca^2+^ oscillations that trigger AMPK activation promote autophagy.

### 3.3. Ca^2+^ oscillations induced AMPK-mediated cellular autophagy

**Figure 5.**
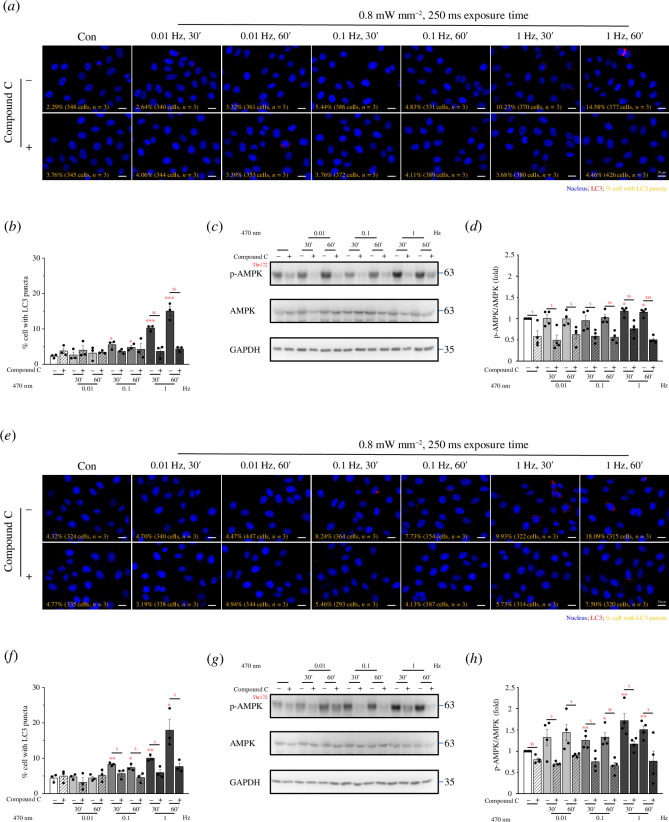
AMPK inhibitor suppresses Ca^2+^-induced cellular autophagy. The cells were pre-treated with Compound C (10 μM) for 30 min and then continuously exposed to blue light at 470 nm (0.8 mW/mm^2^, 250 ms). The frequencies were 0.01, 0.1 and 1 Hz, and the exposure durations were 30 and 60 min. Fluorescence images of (*a*) PANC1-CatCh-Venus and (*e*) U2OS-CatCh-Venus cells displaying LC3 (green) and nuclei (blue) staining. Scale bars: 20 μm. The percentage of cells with LC3 puncta in (*b*) PANC1-CatCh-Venus and (*f*) U2OS-CatCh-Venus cells. p-AMPK, AMPK and GAPDH expression levels were assessed in (*c*) PANC1-CatCh-Venus and (*g*) U2OS-CatCh-Venus cells using Western blotting. Quantitative analysis of the p-AMPK/AMPK relative changes in (*d*) PANC1-CatCh-Venus and (*h*) U2OS-CatCh-Venus cells. Data are shown as means ± s.e.m. based on at least three independent experiments. * *p* < 0.05, ** *p* < 0.01, *** *p* < 0.001, ^$^ *p* < 0.05, ^$$^ *p* < 0.01, ^$$$^ *p* < 0.001. AMP, adenosine monophosphate; AMPK, AMP-activated protein kinase.

AMPK can regulate cellular autophagy, and the experimental data indicated that Ca^2+^ activates AMPK, leading to the initiation of cellular autophagy. Therefore, the AMPK inhibitor Compound C was used to treat CatCh-expressing PANC1 and U2OS cells. The data showed that in cells treated with Compound C, optogenetically engineered Ca^2+^ oscillations did not cause the generation of LC3 puncta or autophagy (figure 5*a*,*b*,*e,f*). In addition, Western blot analysis revealed that Compound C inhibited the increase in AMPK phosphorylation and activation induced by optogenetic stimulation (figure 5*c*,*d*,*g,h*). These results suggest that Ca^2+^ oscillations regulate autophagy through AMPK signalling.

### 3.4. Ca^2+^-triggered cellular autophagy resulting in cell death

**Figure 6.**
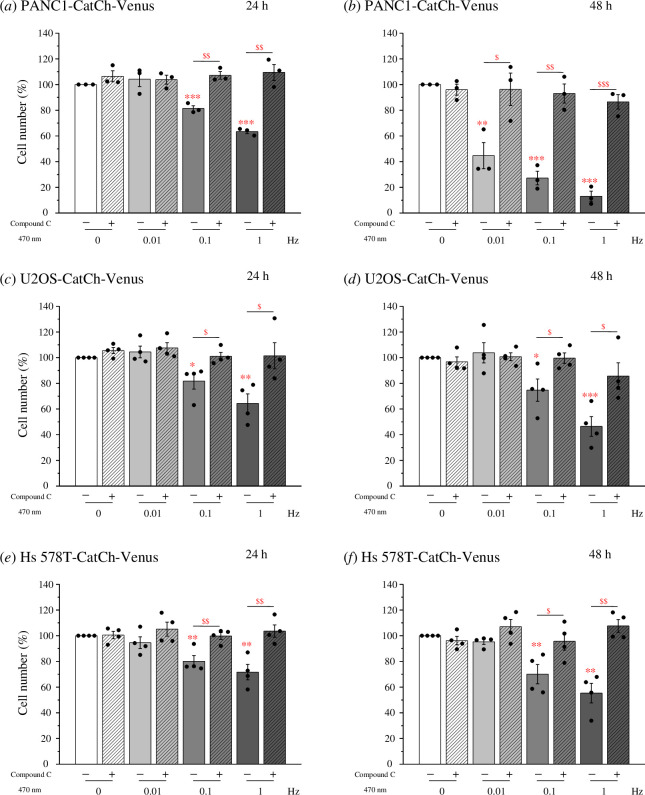
Ca^2+^-triggered AMPK-mediated autophagy leads to cell death. The cells were seeded in 3 cm dishes and incubated overnight. Next, they were pre-treated with 10 μM Compound C for 30 min, followed by optogenetic stimulation using the fixed parameters of 0.8 mW mm^−2^ intensity and 250 ms exposure time, at frequencies of 0.01, 0.1 and 1 Hz for a duration of 60 min. PANC1-CatCh-Venus cells were incubated for (*a*) 24 or (*b*) 48 h before Hoechst staining. U2OS-CatCh-Venus cells were incubated for (*c*) 24 or (*d*) 48 h before Hoechst staining. Hs 578T-CatCh-Venus cells were incubated for (*e*) 24 or (*f*) 48 h before Hoechst staining. Quantitative analysis of cell number. Data are shown as means ± s.e.m. derived from a minimum of three separate experiments. * *p* < 0.05, ** *p* < 0.01, *** *p* < 0.001, ^$^ *p* < 0.05, ^$$^ *p* < 0.01, ^$$$^ *p* < 0.001.

Ca^2+^ induces autophagy and regulates cell survival and death [32,33]. Therefore, we investigated the relationship between Ca^2+^ oscillations, autophagy and cell survival. Optogenetic stimulation using 0.8 mW mm^−2^ intensity, 250 ms exposure time and frequencies of 0.01, 0.1 and 1 Hz was applied. The data showed that high frequency Ca^2+^ oscillation was associated with a lower number of cells, indicating that cell death was induced by Ca^2+^ cytotoxicity (figure 6). This cell death phenomenon was more obvious at 48 h after the cells were exposed to blue light than at 24 h. Furthermore, Compound C and autophagy inhibitors (CQ and 3-MA) treatment almost completely inhibited the cell death caused by light (figure 6, see electronic supplementary material, figure S3). These results suggested that Ca^2+^ oscillations induce AMPK-mediated autophagy and contribute to cell death.

## 4. Discussion

Ca^2+^ plays a crucial role in regulating autophagy in cancer cells [19,34,35]. Previous studies have shown that Ca^2+^ oscillations can stimulate mitophagy, and cellular Ca^2+^ can promote autophagy [36,37]. Therefore, we employed both chemical and optogenetic methods to manipulate intracellular Ca^2+^ concentrations. Using chemical agents, such as ionomycin or TG, to increase intracellular Ca^2+^ levels resulted in an increase in LC3 puncta and autophagy. However, chelation of intracellular Ca^2+^ with BAPTA-AM inhibited LC3 puncta formation (figure 1). This reveals that any increase in extracellular Ca^2+^ influx or ER Ca^2+^ release into the cytoplasm triggers autophagy. Moreover, there was little difference in the degree of autophagy caused by TG in 2 or 0 mM Ca^2+^ culture medium, which shows that the Ca^2+^ released from the ER is sufficient to induce autophagy to a critical point (figure 1). On the other hand, we used optogenetics to modulate different frequencies of Ca^2+^ oscillations. We found that in PANC1 and U2OS cells expressing CatCh, LC3 puncta increased under 0.1 and 1 Hz stimulation (figure 4*a*,*c*). However, an increase in autophagy was observed only under 1 Hz stimulation in Hs 578 T cells expressing CatCh (figure 4*e*), suggesting that a specific threshold of Ca^2+^ is necessary to induce autophagy.

Ca^2+^ can induce cellular autophagy via numerous Ca^2+^-regulated AMPK, DAPK and mTOR pathways [38,39]. In this study, ionomycin and TG increased cytosolic Ca^2+^, leading to AMPK activation. However, no significant changes were examined in DAPK and mTOR activation or expression (figure 3). This suggests that Ca^2+^ induces autophagy via the AMPK pathway. Different Ca^2+^ signalling pathways can activate distinct target proteins, depending on the pattern of Ca^2+^ oscillation [20]. Therefore, the light-sensitive Ca^2+^ channel CatCh was used to explore the influence of Ca^2+^ oscillation frequency on autophagy-related proteins. We demonstrated that AMPK can be activated under light stimulation conditions of 0.1 and 1 Hz but not 0.01 Hz (figure 4*b*,*d,f*). The frequency range of Ca^2+^ oscillations caused by normal physiological stimulation is between 0.1 and 0.01 Hz. This indicated that high-frequency Ca^2+^ oscillations tend to induce AMPK activation. This result is consistent with the frequency range of Ca^2+^ oscillations that can be measured in cells under various autophagy-related stimuli.

Ca^2+^ regulates various cellular functions including proliferation, migration and cell death [40–42]. In our previous studies, optogenetically engineered Ca^2+^ oscillations were used to regulate the activation of Ca^2+^-dependent transcription factors, cell migration, mitochondrial fission and cell death [30,31]. In the present study, we used optogenetics and found that higher-frequency Ca^2+^ oscillations tended to increase cell autophagy and lead to cell death (figure 6). Surprisingly, the AMPK inhibitor almost completely inhibited the autophagy and cell death caused by Ca^2+^ oscillations (figures 5 and 6), indicating that Ca^2+^ regulates cell death by activating AMPK signalling. AMPK plays a crucial role in the induction of autophagy; therefore, Ca^2+^ oscillations induced by optogenetics may contribute to autophagy-mediated cell death.

Cellular life involves four main and closely related fundamental processes: survival, proliferation, differentiation and death. All these processes are intricately associated with Ca^2+^ [22,43–45]. Different Ca^2+^ patterns can trigger distinct Ca^2+^ signalling responses [20]. For instance, sustained Ca^2+^ signals in the ER can lead to cell death, whereas oscillatory Ca^2+^ signals promote cell survival [46]. On the other hand, if mitochondria receive sustained Ca^2+^ signals, it can trigger the production of reactive oxygen species leading to cell apoptosis. However, when cells experience oscillatory Ca^2+^ signalling, they tend to survive [47]. Previous studies have shown that cell death typically requires sustained and high-concentration Ca^2+^ [20,48]. Previous studies have indicated that Ca^2+^ influences AMPK activity; however, the effect of Ca^2+^ oscillations on AMPK activation remains unknown. This study revealed that AMPK activation requires high-frequency Ca^2+^ oscillations, which subsequently lead to cell death through autophagy.

## Data Availability

The data that support the findings of this study are available from the corresponding author upon reasonable request. Electronic supplementary material is available online [49].
